# Temporal dynamics of *RAS* mutations in circulating tumor DNA in metastatic colorectal cancer: clinical significance of mutation loss during treatment

**DOI:** 10.1007/s00432-024-05805-3

**Published:** 2024-05-28

**Authors:** Kenta Iguchi, Manabu Shiozawa, Mamoru Uchiyama, Masahiro Asari, Koji Numata, Yasushi Rino, Aya Saito

**Affiliations:** 1 Department of Colorectal Surgery, Kanagawa Cancer Center, 2-3-2, Nakao, Asahi-ku, Yokohama, 241-8515 Japan; 2 Department of Surgery, Yokohama City University, School of Medicine, Yokohama, Japan

**Keywords:** *RAS* gene, Circulating tumor DNA, Colorectal cancer, Mutation, Prognosis

## Abstract

**Purpose:**

In metastatic colorectal cancer (mCRC), *RAS* mutation loss may occur during the standard-of-care regimen. In this study, we aimed to investigate the temporal dynamics of the *RAS* gene and its clinical significance.

**Methods:**

This was a retrospective, single-center study that included 82 patients with tissue *RAS*-mutant (*RAS*-MT) mCRC who underwent circulating tumor DNA (ctDNA) *RAS* monitoring between January, 2013–April, 2023. Patients were analyzed for the rate of change over time to acquired *RAS* mutation loss (a*RAS*-ML) and clinicopathological factors. The prognostic relevance of mutation loss was assessed.

**Results:**

a*RAS*-ML was detected in 33 (40.2%) patients, 32 of whom had a mutation loss in the first ctDNA *RAS* assay. Patients with a *RAS* mutation detected in the first assay had a median time of 8 months until the second ctDNA *RAS* assay, with 4.5% cases newly converted to a*RAS*-ML; no new conversions were detected at the third assay. The a*RAS*-ML group exhibited more single-organ metastases in the target organ during ctDNA measurement (a*RAS*-ML: 84.8% vs. *RAS*-MT: 59.2%, p = 0.02). Of the 33 patients with a*RAS*-ML, seven (21.2%) received anti-epidermal growth factor receptor (EGFR) therapy, with a median progression-free survival of 8 months. Multivariate analysis revealed that persistent ctDNA *RAS* mutation was an independent prognostic factor for overall survival (hazard ratio: 2.7, 95% confidence interval: 1.1–6.3, p = 0.02).

**Conclusion:**

The rate of ctDNA mutation loss in patients with *RAS*-MT mCRC decreases over time. Therefore, using a ctDNA *RAS* assay early in treatment will assist in challenging the use of EGFR regimens.

**Supplementary Information:**

The online version contains supplementary material available at 10.1007/s00432-024-05805-3.

## Introduction

Colorectal cancer (CRC) is the third most prevalent cancer worldwide and the second leading cause of cancer-related deaths (Keum and Giovannucci 2019). Approximately 150,000 new cases of CRC and 52,580 CRC-related deaths were reported in the United States in 2022 (Siegel et al. 2022). Up to 17.7–22.0% of patients newly diagnosed with CRC acquire metastatic CRC (mCRC), and 19.6–20.6% of patients newly diagnosed with stage I–III CRC eventually develop mCRC (Elferink et al. 2015; Väyrynen et al. 2020; Nors et al. 2023; Siegel et al. 2023; Tsai et al. 2023).

In addition to conventional chemotherapy, several drugs that target the molecular drivers of CRC—such as epidermal growth factor receptor (EGFR) and vascular endothelial growth factor pathways—have been used extensively to improve the survival of patients with mCRC (Cremolini et al. 2015; Venook et al. 2017; Bennouna et al. 2019; Heinrich et al. 2023) Current mCRC treatment guidelines recommend screening for mutations in genes such as *KRAS*, *NRAS*, and *BRAF* before selecting the chemotherapy regimen. *RAS* status is crucial for mCRC treatment decisions (Van Cutsem et al. 2016; Benson et al. 2021; Yoshino et al. 2023). If the *RAS* gene is mutated, certain targeted therapies, such as anti-EGFR agents, may be less effective. Therefore, determining *RAS* status is useful for tailoring treatment plans and increasing the likelihood of favorable outcomes in patients with mCRC.

Recent research suggests that temporal changes may occur in *RAS* status (Osumi et al. 2021; Nicolazzo et al. 2023). In CRC, the condition “Neo*RAS* wild-type (Neo*RAS*-WT)” refers to a situation in which a patient initially had a *RAS* mutation, but after treatment, the status reverted to wild-type (non-mutated). This change can impact treatment decisions because patients with Neo*RAS*-WT tumors may respond differently to certain therapies compared to those with persistent *RAS* mutations (Cremolini et al. 2019; Osumi et al. 2021; Nakajima et al. 2021). Meanwhile, “Neo*RAS*-WT” status remains a controversial concept. In recent years, *RAS* status is being tested using blood samples (Patelli et al. 2021, 2023). The circulating tumor DNA (ctDNA) *RAS* assay, developed using liquid biopsy technology, can reveal inter- and intra-tumor heterogeneity, thereby improving diagnosis by facilitating early detection and screening, monitoring, and follow-up; moreover, personalized therapeutic strategies can improve treatment outcomes (Reinert et al. 2019; Patelli et al. 2023). In a previous study, we reported that the frequency of mutation loss in a single ctDNA *RAS* assay was 43.5% in patients with *RAS* mutations. However, due to dynamic changes in metastatic organs and chemotherapy regimens over the treatment course, it is unclear how *RAS* responds to multiple ctDNA tests over time and what the optimal timing is for ctDNA assay. Determining these parameters is crucial for effective treatment of CRC with *RAS* mutations. Therefore, in this study, we aimed to determine the temporal dynamics of *RAS* status during treatment and its clinical significance using ctDNA *RAS* assay in patients with mCRC harboring mutant *RAS*.

## Methods

### Study population

This single-center retrospective cohort study was conducted at the Kanagawa Cancer Center, Yokohama, Japan. The study was conducted in accordance with the principles of the Declaration of Helsinki and was approved by the Institutional Review Board of Kanagawa Cancer Center (Approval Number: 2023-85). The requirement for written informed consent was waived due to the retrospective nature of the study, and participants were given the opportunity to opt-out. The study was registered in the Japanese Clinical Trials Registry, with registration number UMIN000053017. Consecutive patients who had *RAS* mutant-type (*RAS*-MT) mCRC between January 1, 2013, and April 30, 2023, were included in this study and their *RAS* status was monitored. Patient data, including demographic, clinical, and pathological information, were extracted from their medical records. Patients who met the following inclusion criteria were enrolled: (1) histologically diagnosed with adenocarcinoma, and (2) *RAS*-MT (*KRAS/NRAS* exon 2, 3, or 4) confirmed in primary tumor tissue before beginning chemotherapy. Patients with colon cancer were excluded from this study.

### Testing and monitoring of RAS genotypes

To determine *RAS* status before starting treatment, tumor tissue taken surgically or by biopsy from the primary lesion was tested using the MEBGEN RASKET^™^-B kit (Medical & Biological Laboratories Co., Tokyo, Japan). Genomic DNA was extracted from formalin-fixed paraffin-embedded tissue sections, and the DNA concentrations ranged from 10 to 20 ng/µL. Polymerase chain reaction was performed with reverse sequence-specific oligonucleotides for all the samples. We examined the presence of the following mutations for exon 2 (G12S, G12C, G12R, G12D, G12V, G12A, G13S, G13C, G13R, G13D, G13V, and G13A), exon 3 (A59T, A59G, Q61K, Q61E, Q61L, Q61P, Q61R, and Q61H), and exon 4 (K117N, A146T, A146P, and A146V) of *KRAS* and *NRAS*.

After the initiation of standard-of-care regimens, the OncoBEAM^™^ RAS CRC assay (Sysmex Inc., Kobe, Japan) was used to monitor *RAS* mutations over time. The OncoBEAM^™^ *RAS* assay is a molecular diagnostic test designed to detect mutations in *RAS* genes, specifically *KRAS* and *NRAS*. This assay uses liquid biopsy technology, whereby ctDNA shed by cancer cells into the plasma is analyzed. During the course of treatment, ctDNA was assayed with OncoBEAM^™^ at the discretion of the physician. Before the ctDNA *RAS* assay, the patients were treated with standard-of-care regimens. The use of anti-EGFR agents for patients with a*RAS*-ML by ctDNA *RAS* assay was decided on a case-by-case basis. Clinicopathological background, including age, sex, tumor location, and treatment regimen, was compared between patients who acquired *RAS* mutation loss (a*RAS*-ML) during treatment and those who retained a *RAS* mutation. The rate of conversion to a*RAS*-ML over time was analyzed, and the prognostic impact of mutation loss and optimal testing time was assessed.

### Definition of aRAS-ML

In this study, a*RAS*-ML was defined as the absence of *RAS* mutations after treatment initiation in patients who were diagnosed with *RAS*-MT (*KRAS* exons 2, 3, 4 or *NRAS)* upon pre-treatment tissue examination; mutation loss was determined based on the findings of the transient OncoBEAM^™^ RAS CRC assay. Patients with a mutation detected in the first ctDNA *RAS* assay were tested again during the treatment course, and were classified as a*RAS*-ML if no *RAS* mutation was detected in subsequent tests.

### Statistical analysis

Continuous variables were analyzed using the Mann–Whitney U test and described using the median and interquartile range. Differences between categorical variables were analyzed using Fisher’s exact probability test. Survival outcomes were assessed using the Kaplan–Meier method, and group data were compared using the log-rank test. A multivariate Cox proportional hazard model was used to analyze the impact of acquired mutation loss on survival. Factors significant for overall survival in the univariate analysis were entered into the model using the forced entry method. Statistical analyses were carried out using EZR (Saitama Medical Center, Jichi Medical University, Saitama, Japan), a graphical user interface for R (The R Foundation for Statistical Computing, Vienna, Austria), with a significance level of p < 0.05.

## Results

### Patient characteristics

A total of 82 patients with *RAS*-MT mCRC (median age: 66 years; range: 26–86 years) were eligible for this study. Of these, 33 patients (40.2%) acquired mutation loss on *RAS* during the treatment course. The clinicopathological characteristics of the patients are listed in Table 1. No differences were observed between the a*RAS*-ML and *RAS*-MT groups in terms of patient background, including age, sex, and tumor location.

**Table 1:** Demographic and clinicopathological characteristics of patients with metastatic colorectal cancer

	*RAS*-MT (*n* = 49)	a*RAS*-ML (*n* = 33)	*P*-value
Age (years)			
Median (IQR)	66 (53–74)	65 (54–72)	0.9
70 ≤ , n (%)	15 (30.6)	11 (33.3)	0.8
70 > , n (%)	34 (69.4)	22 (66.7)	
Sex, n (%)			0.3
Male	28 (57.1)	15 (45.5)	
Female	21 (42.9)	18 (54.5)	
Histological type, n (%)			0.6
Differentiated	48 (98.0)	31 (93.9)	
Poorly differentiated	1 (2.0)	2 (6.1)	
Tumor sidedness, n (%)			0.6
Right sided	13 (26.5)	7 (21.2)	
Left sided	36 (73.5)	26 (78.8)	
Microsatellite instability, n (%)			1
MSS	48 (98.0)	33 (100)	
MSI-H	1 (2.0)	0 (0)	
Resection of primary tumor, n (%)			0.3
Yes	46 (93.9)	33 (100)	
No	3 (6.1)	0 (0)	
Distant metastasis at the start of standard-of-care regimen, n (%)			
Liver			0.2
Present	29 (59.2)	14 (42.4)	
Absent	20 (40.8)	19 (57.6)	
Lung			0.8
Present	20 (40.8)	15 (45.5)	
Absent	29 (59.2)	18 (54.5)	
Peritoneum			0.4
Present	10 (20.4)	4 (12.1)	
Absent	39 (79.6)	29 (87.9)	
Lymph node			0.7
Present	4 (8.2)	4 (12.1)	
Absent	45 (91.8)	29 (87.9)	
Local			1
Present	2 (4.1)	2 (6.1)	
Absent	47 (95.9)	31 (93.9)	
Bone			1
Present	1 (2.0)	0 (0)	
Absent	48 (98.0)	33 (100)	
Ovary			0.4
Present	0 (0)	1 (3.0)	
Absent	49 (100)	32 (97.0)	

*MT* mutant type, *aRAS-ML* acquired *RAS* mutation loss,

*IQR* interquartile range, *MSS* microsatellite stable, *MSI-H* microsatellite instability-high

At the beginning of the standard-of-care regimen, the most common target organs were the lungs, liver, peritoneum, and lymph nodes in the a*RAS*-ML group, and the liver, lungs, and peritoneum in the *RAS*-MT group. The most common *RAS* mutation found in pre-treatment tissue was *KRAS* codon 12, in both the a*RAS*-ML and *RAS*-MT groups (supplement).

### Results of plasma ctDNA RAS monitoring

**Fig. 1:**
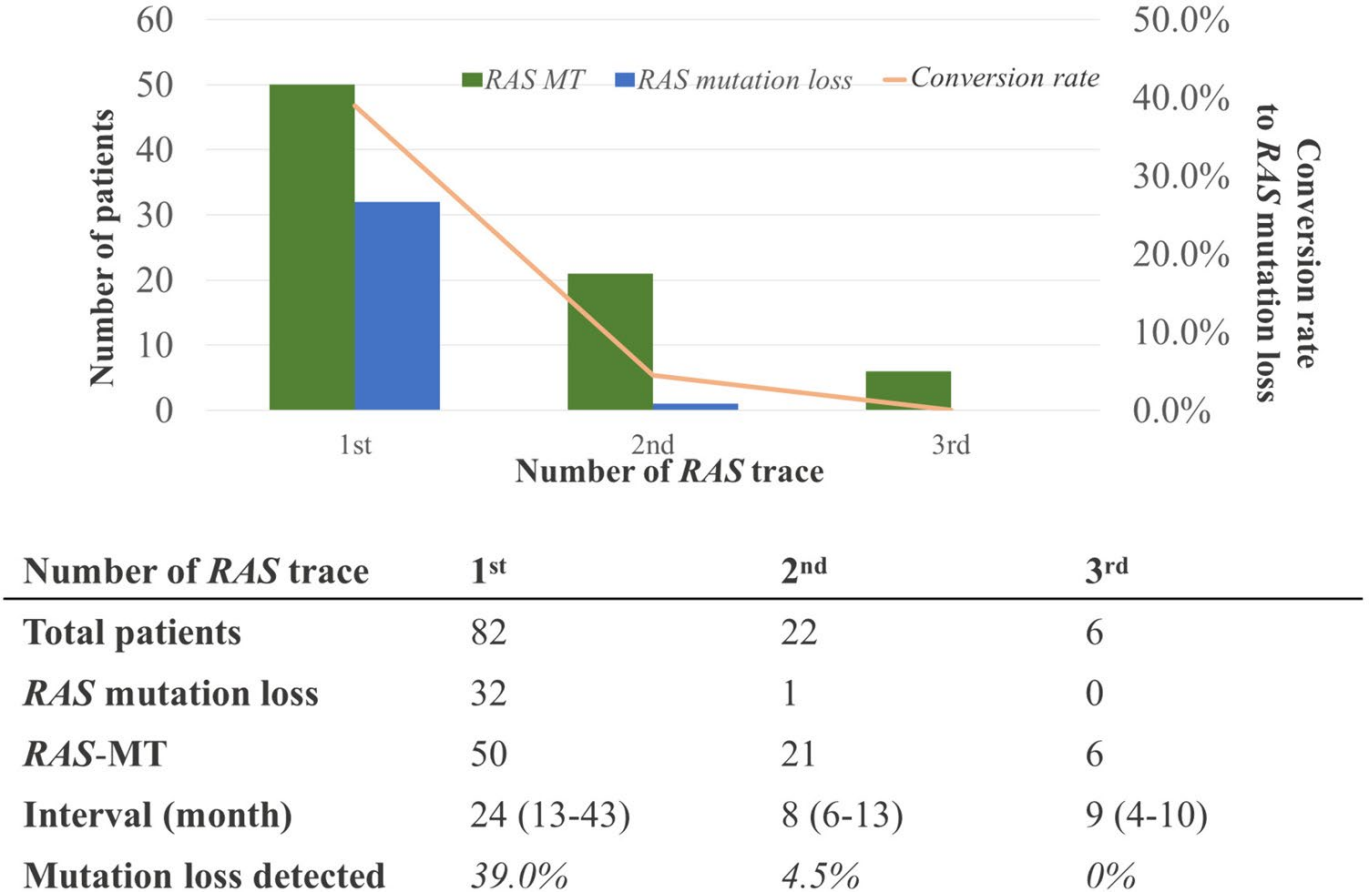
Temporal diversity of *RAS* mutations

The median time between tissue *RAS* testing and the first ctDNA *RAS* assay was 24 months; 21 months (range: 16–43 months) in the a*RAS*-ML group and 25 months (range: 15–34 months) in the *RAS*-MT group, with no significant differences (p = 0.73). a*RAS*-ML was detected in 32 (39.0%) patients during the first ctDNA *RAS* assay. A second ctDNA *RAS* assay was performed in 22 patients who were shown to have a *RAS* mutation in the first assay, with one patient (4.5%) having a*RAS*-ML. In this case, the interval between the first and second ctDNA assays was 11 months. A third ctDNA assay was performed in six patients, and *RAS* mutation was detected in all cases. In addition, ctDNA assays were performed in all patients detected to harbor a*RAS*-ML at the time of the first- or second-line standard regimens, and no patients were detected to harbor a*RAS*-ML after the third-line regimen or later (Fig. 1). A trend towards increasing single-organ metastases in the target organ for treatment was observed in the a*RAS*-ML group at the first ctDNA assay, when compared with that in the *RAS*-MT group (84.8% vs. 59.2%, p = 0.02) (Table 2). There was no trend in *RAS* mutation loss according to the type of chemotherapy regimen administered. Among the patients with a*RAS*-ML, seven patients (21.2%) received anti-EGFR therapy after mutation loss and the median progression-free survival was 8 months (range: 4–12 months). Of these seven patients, six (85.7%) had mutations detected in the ctDNA *RAS* assay after treatment. There was no significant difference in overall survival between those who received anti-EGFR therapy and those who did not in patients with a*RAS*-ML (p = 0.8).

**Table 2:** Summary of treatment course in patients with *RAS* mutations and patients without mutations

	*RAS*-MT (*n* = 49)	a*RAS*-ML (*n* = 33)	*P*-value
Treatment details for systemic therapy at the time of ctDNA *RAS* assay^a^, n (%)			
Triplet + aVEGF/Triplet^b^	19 (38.8)	16 (48.5)	0.5
Doublet + aVEGF/Doubet^c^	23 (46.9)	17 (51.5)	0.8
Capecitabine + aVEGF	1 (2.0)	0 (0)	1
Trifluridine / tipiracil + aVEGF	2 (4.1)	0 (0)	0.5
Regorafenib	3 (6.1)	0 (0)	0.3
Nivolmab	1 (2.0)	0 (0)	1
Treatment line at the time of ctDNA *RAS* assay, n (%)			0.4
1st line	30 (61.2)	21 (63.6)	
2nd line	14 (28.6)	12 (36.4)	
3rd line	3 (6.1)	0 (0)	
4th line	2 (4.1)	0 (0)	
Target lesion at the first time of ctDNA *RAS* assay, n (%)			
Multi-organ/Single-organ	20 (40.8)/29 (59.2)	5 (15.2)/28 (84.8)	0.02
Liver (present/absent)	29 (59.2)/20 (40.8)	14 (42.4)/19 (57.6)	0.2
Lung (present/absent)	20 (40.8)/29 (59.2)	16 (48.5)/17 (51.5)	0.5
Peritoneum (present/absent)	12 (24.5)/37 (75.5)	4 (12.1)/29 (87.9)	0.3
Lymph node (present/absent)	3 (6.1)/46 (93.9)	2 (6.1)/31 (93.9)	1
Local (present/absent)	0 (0)/49 (100)	2 (6.1)/31 (93.9)	0.2
Bone (present/absent)	4 (8.2)/45 (91.8)	1 (3.0)/32 (97.0)	0.6
Ovary (present/absent)	0 (0)/49 (100)	0 (0)/33 (100)	1

*MT*, mutant type; *aRAS-ML*, acquired *RAS* mutation loss

^a^Chemotherapy or immune checkpoint inhibitors

^b^*Triplet*; FOLFOXIRI (infusional fluorouracil, leucovorin, oxaliplatin, and irinotecan) or CAPOXIRI (capecitabine in combination with irinotecan and oxaliplatin)

^c^*Doublet*; FOLFOX (infusional fluorouracil, leucovorin, and oxaliplatin), FOLFIRI (infusional fluorouracil, leucovorin, and irinotecan), IRIS (S-1 in combination with irinotecan), or SOX (S-1 in combination with oxaliplatin)

### Relationship between aRAS-ML and prognosis

**Fig. 2:**
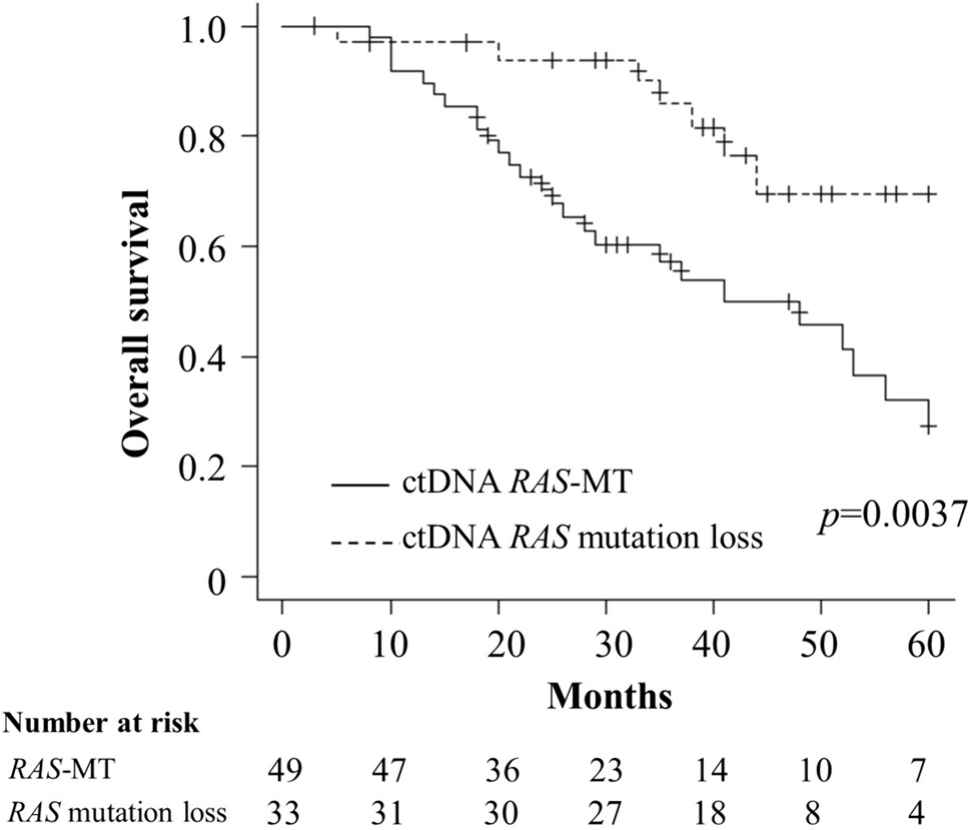
Overall survival with acquired *RAS*-mutation loss (a*RAS*-ML) versus ctDNA *RAS*-MT

The median follow-up duration was 35 months. The 3-year overall survival rate was significantly better in the a*RAS*-ML group (85.9%) compared with that in the *RAS*-MT group (57.2%; p = 0.0037) (Fig. 2). Table 3 shows the results of univariate analysis of overall survival for each clinicopathologic factor. Multivariate analysis showed that persistent ctDNA *RAS* mutation during the treatment was an independent prognostic factor for overall survival (hazard ratio: 2.7, 95% confidence interval: 1.1–6.3, p = 0.02) (Table 4).

**Table 3:** Analysis of overall survival by clinicopathological factors in metastatic colorectal cancer with *RAS* mutation

Variables	*n* (%)	3y-OS	95% CI	*P*-value
Sex				
Male	43 (52.4)	0.67	0.5–0.8	0.6
Female	39 (47.6)	0.70	0.5–0.8	
Age				
70 >	56 (68.3)	0.69	0.5–0.8	0.3
70 ≤	26 (31.7)	0.69	0.4–0.8	
Tumor sidedness				
Right sided	20 (24.4)	0.62	0.4–0.8	0.1
Left sided	62 (75.6)	0.71	0.6–0.8	
Distant metastasisat the start of chemotherapy				
Liver				
Present	43 (52.4)	0.64	0.5–0.8	0.1
Absent	39 (47.6)	0.75	0.6–0.9	
Lung				
Present	35 (42.7)	0.77	0.6–0.9	0.3
Absent	47 (57.3)	0.63	0.5–0.8	
Peritoneum				
Present	14 (17.1)	0.60	0.3–0.8	0.09
Absent	68 (82.9)	0.71	0.6–0.8	
Lymph node				
Present	8 (9.8)	0.88	0.4–0.98	0.4
Absent	74 (90.2)	0.67	0.5–0.8	
Local				
Present	4 (4.9)	0.75	0.1–0.96	0.6
Absent	78 (95.1)	0.68	0.6–0.8	
Ovary				
Present	1 (1.2)	< NA >	< NA >	0.3
Absent	81 (98.8)	0.70	0.6–0.8	
Bone				
Present	1 (1.2)	< NA >	< NA >	0.0002
Absent	81 (98.8)	0.70	0.6–0.8	
Target lesion at the first time of ctDNA *RAS* assay				
Multi-organ	25 (30.5)	0.58	0.4–0.7	0.003
Single-organ	57 (69.5)	0.74	0.6–0.8	
ctDNA *RAS* status				
a*RAS*-ML	33 (40.2)	0.86	0.7–0.9	0.004
*RAS*-MT	49 (59.8)	0.57	0.4–0.7	

*OS* overall survival rate, *CI* confidence interval,* MT* mutant type, *aRAS-ML* acquired *RAS* mutation loss,* ctDNA* circulating tumor DNA

**Table 4:** Multivariate analysis of predictors of overall survival

	Hazard ratio	95% confidence interval (lower–upper)	*P*-Value
Presence of bone metastases			
Present	9.6	1.0–87.8	0.045
Absent	1		
Target lesion at the first time of ctDNA RAS assay			
Multi-organ	2.1	1.0–4.2	0.04
Single-organ	1		
ctDNA RAS status			
*RAS*-MT	2.7	1.1–6.3	0.02
a*RAS*-ML	1		

*ctDNA* circulating tumor DNA,* MT* mutant type, *aRAS-ML* acquired *RAS* mutation loss

## Discussion

The present study revealed the temporal dynamics of *RAS* in mCRC. To the best of our knowledge, this is the first study to analyze and report the long-term changes in *RAS* and a*RAS*-ML detection rate. We showed that the detection rate of a*RAS*-ML decreased during the course of treatment. Moreover, our results indicate that patients with mutation loss may have better prognosis than patients with persistent *RAS* mutations.

The outcome of ctDNA *RAS* testing is a valuable indicator for challenging anti-EGFR antibody treatment in patients with *RAS*-WT tissues (Cremolini et al. 2019; Patelli et al. 2023). The CHRONOS trial, a phase 2 study that reported the results of anti-EGFR antibody rechallenge following ctDNA evaluation, reported partial responses in 30% and disease control in 63% cases with *RAS*-WT status (Sartore-Bianchi et al. 2022). The VELO trial reported by Napolitano et al. analyzed plasma ctDNA in patients with CRC having *RAS/BRAF*-WT before treatment; in their study, the median progression-free survival was 4.5 months in the panitumumab plus trifluridine-tipiracil group (95% confidence interval: 2.2–6.8 months) vs. 2.6 months in the trifluridine–tipiracil only group (95% confidence interval: 1.0–4.3 months) (Napolitano et al. 2023). Thus, although some studies have reported EGFR antibody rechallenge using ctDNA assays in recent years, the clinical significance of a*RAS*-ML in mCRC is not well established. Mutation loss after induction chemotherapy in patients with *RAS*-MT was between 21.5 and 90% (Klein-Scory et al. 2020; Sunakawa et al. 2022; Wang et al. 2022; Osumi et al. 2023).

Sunakawa et al. reported the rate of early disappearance of *RAS* mutations in patients receiving FOLFOXIRI plus bevacizumab as first-line treatment for CRC, as monitored by performing liquid biopsy (Sunakawa et al. 2022). After 8 weeks of treatment, they observed a *RAS*-MT disappearance rate of 78% based on ctDNA testing results. In the present study, there was a lower frequency of *RAS* mutation loss in more late-line treatment and fewer cases of mutation loss in patients with multi-organ metastases. It can be assumed that more cases with multi-organ metastases occur in late-line treatment due to disease progression, and that the frequency of mutation loss is lower due to tumour heterogeneity caused by multi-organ metastases, rather than the type of regimen. In view of this, it is possible that a more aggressive regimen at an earlier stage of treatment, with a reduction in the number of target lesions, may reduce tumour heterogeneity and result in a*RAS*-ML, thus improving the long-term prognosis. The higher rate of mutation loss observed in the report by Sunakawa et al. with the potent regimen FOLFOXIRI plus bevacizumab may provide support for this hypothesis.

A clinical problem with standard systemic regimens for mCRC is the oncological heterogeneity of target metastases (Kim et al. 2015; Lu et al. 2016; Adua et al. 2017; Morris and Strickler 2021; Nicolazzo et al. 2021). In addition to differences in the metastatic organs such as the liver, lung, lymph nodes, and peritoneum, the heterogeneity of multiple metastases within an organ can lead to inconsistent chemotherapy efficacy and indications. Unlike conventional organ biopsies, liquid biopsy testing for *RAS* mutations may represent the overall disease status. Therefore, ctDNA testing by liquid biopsy in mCRC may reflect the disease status of mCRC in real-time, considering the heterogeneity of multiple metastases. Our findings showed that the presence or absence of a*RAS*-ML status (as determined by temporal ctDNA testing) may be a prognostic marker. Hence, monitoring the status of *RAS* mutations during the course of treatment may help to identify groups of patients with a better prognosis.

In this study, loss of *RAS* mutation was an independent prognostic factor. Additionally, the a*RAS*-ML group tended to have more cases with a single target organ at the time of ctDNA collection. This suggests that the disappearance of the *RAS* mutation in the blood may be a marker of the therapeutic efficacy in patients with tissue *RAS*-MT. In mCRC, tumor heterogeneity within each metastatic lesion or within a metastatic tumor may lead to differential response to standard chemotherapy (Adua et al. 2017; Wang et al. 2022). This is supported by the higher incidence of single-organ metastases in the a*RAS*-ML group in our study. Oncological heterogeneity may be higher in cases of multiple-organ metastases than in cases of single-organ metastases. Therefore, if at the time of ctDNA *RAS* testing, a patient shows single-organ metastasis, *RAS*-MT clearance may be increased, resulting in a*RAS*-ML. In other words, patients with single-organ metastases are more likely to have a*RAS*-ML and should be actively monitored, and the possibility of EGFR antibody challenge should be considered. Meanwhile, there is currently no evidence to suggest that anti-EGFR agents has any benefit for patients with a*RAS*-ML. The C-PROWESS study, a phase II trial evaluating the safety and efficacy of panitumumab in combination with irinotecan for the management of Neo*RAS*-WT colorectal cancer, is currently underway in Japan and the results of this trial are awaited(Osumi et al. 2022). Challenges related to assessing difficult-to-image metastases, such as micro-metastases, and the lack of evidence for the therapeutic efficacy of EGFR antibodies in a*RAS*-ML remain to be overcome in the future studies.

The Neo*RAS*-WT concept refers to the loss of detectable *RAS* mutation in the presence of continued evidence of mCRC. The present study analyzed the temporal dynamics of the ctDNA *RAS* and suggests that it may serve as a marker for treatment response. When patients respond to treatment, all ctDNA level decrease and, in the best responses, become undetectable. For ctDNA to be in a biological Neo*RAS*-WT state, it must remain detectable at a level where the *RAS* mutation can be detected. Therefore, this marker may be associated with improved survival as it identifies patients who respond well to treatment. Consistent with this, all patients who had a third assessment in the later stages of treatment had detectable *RAS* mutation, and no patients had mutation loss at the third assessment, suggesting a rebound in detection with an increase in tumor volume as treatment becomes less effective. In other words, the ctDNA *RAS* assay may not show a loss of chemotherapy-induced *RAS* mutation, but rather a decrease in the detection of ctDNA. It is important to note that the concept of Neo*RAS*-WT may differ from the clinical significance of loss of ctDNA *RAS* mutation loss during treatment. Meanwhile, temporal *RAS* status is also important in the decision to use EGFR therapies and may therefore be a significant marker.

A notable issue regarding the characterization of Neo*RAS*-WT revolves around the inherent challenge in detecting loss of *RAS* mutation or the absence of detectable ctDNA. As a result, ascertaining whether ctDNA could not be detected owing to a diminutive tumor volume and inability to determine metastatic involvement in specific organs remains a significant hurdle. Osumi et al. pointed out that conversion to Neo*RAS*-WT may be more common in patients without liver metastases (Osumi et al. 2023). Neo*RAS*-WT is defined as the absence of *RAS* mutation coupled with trunk mutations of genes such as TP53, APC, or methylated genes (Moati et al. 2020; Nicolazzo et al. 2020, 2021). Nicolazzo et al. reported that the most common actionable mutations detected in patients with Neo*RAS*-WT were *PIK3CA* (35.7%), *RET* (11.9%), *IDH1* (9.5%), *KIT* (7%), *EGFR* (7%), *MET* (4.7%), *ERBB2* (4.7%), and *FGFR3* (4.7%) (Nicolazzo et al. 2023). The prevailing notion is that concurrent genetic alterations serve as corroborative evidence for the presence of tumor DNA in the plasma. Hence, further investigations in a*RAS*-ML cases utilizing next-generation sequencing, methylated genes, or other serum tumor markers are imperative to clarify this; it is one of the limitations of our present study. Furthermore, a method remains to be established for reliable identification of tumor-derived ctDNA in blood.

The present study has some other limitations. This is a retrospective study with a small sample size; therefore, the treatment course and standard-of-care regimens were not standardized. Additionally, the study did not examine the impact of chemotherapy response (i.e. partial response, stable disease or progressive disease) on ctDNA *RAS* status. Further investigation is required to determine whether differences in chemotherapy response are associated with differences in *RAS* mutation loss rates. Larger clinical trials are required to determine the optimal time for testing ctDNA *RAS*, the choice of regimen, the choice of treatment line (i.e., first-line, second-line, third-line, or later), and how to detect the presence of tumor-derived ctDNA. The importance of anti-EGFR agents in *RAS*-WT mCRC is further strengthened by the results of the PARADIGM trial(Watanabe et al. 2023). In daily clinical practice, mCRCs that are *RAS*-MT at the start of treatment and convert to a*RAS*-ML during the course of treatment may challenge anti-EGFR agents that were previously considered to be of no benefit in *RAS*-MT colorectal cancer, potentially significantly influencing treatment strategies. As each case of mCRC varies significantly in terms of tumor volume, metastatic organs, and other parameters, several challenges persist for “standardizing”. In this regard, tracking ctDNA *RAS* mutations by liquid biopsy may be useful to determine overall tumor status.

## Conclusions

The rate of ctDNA mutation loss in patients with *RAS*-MT mCRC decreases over time. Therefore, ctDNA *RAS* assay should be performed early in treatment to challenge the use of EGFR regimens.

## Supplementary Information

Below is the link to the electronic supplementary material.

Supplementary file1 (DOCX 22 KB)

## Data Availability

The data that support the findings of this study are available on request from the corresponding author. The data is not publicly available because it contains information that could compromise the privacy of research participants.

## Abbreviations

CRC: Colorectal cancer
ctDNA: Circulating tumor DNA
EGFR: Epidermal growth factor receptor
mCRC: Metastatic colorectal cancer
NeoRAS-WT: NeoRAS wild-type
RAS-MT: RAS-Mutant
aRAS-ML: RAS mutation loss
