# Supplementary material for: Temporal dynamics of RAS mutations in circulating tumor DNA in metastatic colorectal cancer: clinical significance of mutation loss during treatment
Source: J Cancer Res Clin Oncol. 2024 May 28;150(5):281. doi: 10.1007/s00432-024-05805-3 (PMC11133214; doi:10.1007/s00432-024-05805-3)
Supplement: Supplementary file 1 — Supplementary file1 (DOCX 22 KB) [file 432_2024_5805_MOESM1_ESM.docx]

**Supplement Table 1.**  Relationship between tissue *RAS* mutations and ctDNA *RAS*

| ***Tissue RAS*** | ***RAS*-MT**  **(n=49)** | **a*RAS*-ML**  **(n=33)** | **P-value** |
| --- | --- | --- | --- |
| ***KRAS* exon2, n (%)** | 45 (91.8) | 28 (84.8) | 0.5 |
| Codon12 | 36 (73.5) | 24 (72.7) | 1 |
| G12A | 4 (8.2) | 1 (3.0) |  |
| G12C | 5 (10.2) | 2 (6.1) |  |
| G12D | 12 (24.5) | 12 (36.4) |  |
| G12R | 1 (2.0) | 0 (0) |  |
| G12S | 2 (4.1) | 3 (9.1) |  |
| G12V | 11 (22.4) | 5 (15.2) |  |
| Subtype unknown | 1 (2.0) | 1 (3.0) |  |
| Codon13 | 9 (18.4) | 4 (12.1) | 0.6 |
| G13D | 9 (18.4) | 4 (12.1) |  |
| ***KRAS* exon3, n (%)** | 2 (4.1) | 4 (12.1) | 0.2 |
| Codon61 | 2 (4.1) | 4 (12.1) | 0.2 |
| Q61H | 1 (2.0) | 3 (9.1) |  |
| Q61L | 1 (2.0) | 1 (3.0) |  |
| ***KRAS* exon4, n (%)** | 1 (2.0) | 0 (0) | 1 |
| Codon117 | 1 (2.0) | 0 (0) | 1 |
| K117N | 1 (2.0) | 0 (0) |  |
| ***NRAS* exon3, n (%)** | 1 (2.0) | 1 (3.0) | 1 |
| Codon61 | 1 (2.0) | 1 (3.0) | 1 |
| Q61L | 1 (2.0) | 0 (0) |  |
| Q61K | 0 (0) | 1 (3.0) |  |

*MT*, mutant type; *aRAS-ML*, acquired *RAS* mutation loss
